# Qualitative exploration of sexual life among breast cancer survivors at reproductive age

**DOI:** 10.1186/s12905-021-01212-9

**Published:** 2021-02-09

**Authors:** Maryam Maleki, Abbas Mardani, Mansour Ghafourifard, Mojtaba Vaismoradi

**Affiliations:** 1 Department of Nursing, School of Nursing and Midwifery, Shahroud University of Medical Sciences, Shahroud, Iran; 2 Nursing Care Research Center, School of Nursing and Midwifery, Iran University of Medical Sciences, Tehran, Iran; 3 Department of Medical‐Surgical Nursing, Faculty of Nursing and Midwifery, Tabriz University of Medical Sciences, Tabriz, Iran; 4 Faculty of Nursing and Health Sciences, Nord University, Bodø, Norway

**Keywords:** Breast cancer, Cancer survivors, Sexual dysfunction, Sexual life, Qualitative research, Quality of care

## Abstract

**Background:**

Our understanding of the experiences of women at reproductive age regarding sexual life and issues they may face after starting the treatment of breast cancer is limited. Therefore, this qualitative study aimed to explore sexual life and its related issues among breast cancer survivors at reproductive age in Iran.

**Methods:**

A qualitative research was conducted. Participants were 21 breast cancer survivors who were under 51 years of age that were chosen using purposeful sampling. In-depth semi-structured interviews were carried out for data collection and the content analysis method was used for data analysis.

**Results:**

The data analysis led to the development of main theme of ‘unfulfilled sexual life’. Also, four subthemes were: ‘undesirable sexual function’, ‘context-based beliefs’, ‘unmet information and supportive needs’, and ‘emotional crisis’.

**Conclusions:**

Our research findings inform healthcare providers about the experiences of breast cancer survivors and related changes in their sexual and marriage life at reproductive age. Nurses and other healthcare providers in the multidisciplinary team should proactively identify health-related problems and design appropriate caring strategies to mitigate sexual and marriage issues among breast cancer survivors. Also, the establishment of sexual health counseling units for breast cancer survivors can help this vulnerable group of women with the improvement of their long-term sexual satisfaction.

**Supplementary Information:**

The online version contains supplementary material available at 10.1186/s12905-021-01212-9.

## Background

Breast cancer (BC) is the most common diagnosed cancer among women, accounting for 11.6% of all cancers across the globe [1]. It has been shown that the prevalence rate of BC is growing quickly in developing countries [1]. According to the World Health Organization (WHO), BC has a prevalence rate of 12.5% among all cancers and is the most common type of cancer among Iranian women [2]. Although the five-year survival rate of BC has reached above 80% in developed countries, the condition is worse in developing countries due to limited healthcare resources and delay in diagnosis [3].

The prevalence of BC among Iranian women is increasing continuously and it is more common in women under the age of 40 years [4]. Therefore, the quality of life (QoL) of these women consisting of their sexual function is mainly influenced by the diagnosis and treatment of BC [5]. Sexual function is a fundamental aspect of patients’ wellbeing, because sexual issues can impact on their QoL and persist into the longer term after finishing BC treatment [6].

Treatment modalities for BC can lead to various sexual problems among BC survivors [7]. For example, mastectomy can stimulate intense emotional stress, change self-perception and body image, and reduce sexual sensitivity due to losing sensations in breasts [8]. Chemotherapy can lead to premature menopause and radiotherapy can cause painful dermatitis and both reduce sexual desire in these women [9]. Treatment-related changes can decrease libido, or cause sexual arousal disorders, dyspareunia and failure to reach orgasm, and eventually reduce sexual activity and sexual satisfaction [10].

BC may further impact on marital relationships, because sexual relationship is one of the main aspects of a successful marital life [11]. Approximately, 61.1% of women with BC report that their sexual relationships with their spouses have been worsened after starting treatment [12]. However, in a study on metastatic BC, Milbury and Badr found that sexual intercourse during cancer treatment can help couples maintain connection and closeness, decrease psychological stress experienced during cancer, and help the couples cope with new life situations [13]. It has been reported that couples affected by BC often experience changes in their sexual relationships and report various sexual problems related to cancer [14]. Some women with BC may decide to continue sexual relationships with their husbands, because of the feeling of love for their family, despite the experience of pain and suffering. Others may agree with their spouse to maintain a close and intimate relationship with each other, but they may avoid having vaginal intercourse for various unknown reasons [15].

## Background in Iran

In the Iranian cultural with a Muslim context, women often are passive during their sexual relationships and sexual request is male-centered. Indeed, the power imbalance in the family relationship contributes to less attention to women’s sexual satisfaction and need [16]. Therefore, satisfying the male partner is the dominant feature of the sexual relationship [17]. Also, couples often do not talk to each other about their sexual desires due to the feeling of shame, which hinders reaching agreements on how to resolve sexual issues [9]. Also, social shame hinders them to discuss about their sexual health issues with strangers including doctors and nurses, and their sexual health issues remain unidentified [18]. It is noted that the Iranian healthcare system has not reached the optimal level of quality and performance to help with sexual problem-solving for couples with chronic diseases and cancer [19]. For instance, no consultation or training regarding how to tackle sexual problems during BC is provided [20]. Therefore, women with BC have many ambiguities and often have to rely on informal sexual health information suggested by others [19].

Improving our understanding of sexual function among women with BC is needed to improve their sexual health and QoL. Since quantitative methods usually are unable to fully reveal the complexities and cultural nature of married sexual life [21], the present study used qualitative methodology aimed to explore sexual life and its related issues among BC survivors at reproductive age in Iran.

## Methods

### Design and participants

This qualitative study used a content analysis approach to investigate sexual life and related issues among BC survivors at reproductive age living in an urban area of Iran. As a qualitative descriptive research method, content analysis provides new insights into the study phenomenon using the condensation and abstraction of textual data [22, 23].

Inclusion criteria to recruit participants were: age under 51 years as the mean age of menopause among Iranian women [24, 25], being married, stage 1–3 of BC based on the patient’s health file, having completed BC treatments within the past 1 to 5 years, and lack of mental and other chronic diseases. Accordingly, the participants were recruited through the purposive sampling method from June 2019 to February 2020.

To identify the potential participants, the medical file of BC survivors who had completed their treatments in the last 1 to 5 years in a referral hospital was reviewed by two researchers (MM, AM). Next, those BC survivors who had the inclusion criteria were contacted via making phone calls and were invited to take part in the study. Sampling continued until data saturation was achieved and no new finding was generated after the analysis of data collected from 21 BC survivors.

The standards for reporting qualitative research (SRQR) guideline was used for reporting this study [26].

### Ethics considerations

The protocol of this study was approved by the Ethics Committee of the University of Medical Sciences in which the first author (MM) worked under the code of IR.SHMU.REC.1398.012. Prior to the study, the participants were fully informed about the research purpose, voluntary participation, their anonymity and confidentiality of collected data. The participants signed a written informed consent form and gave the permission to audio-record the interviews before entering the study.

### Data collection

In-depth semi-structured interviews using open-ended questions were conducted at participants’ preferred times and places so that  they did not interfere with their daily life routines. The interviews were conducted using an interview guide in Farsi by the female researcher (MM) who was a qualified researcher in the field of oncology nursing. The interview guide was developed based on the researchers’ experiences and after performing a review of literature that covered sexual life and related issues among BC survivors (Additional file 1). It included the following questions: ‘how have your sexual experiences changed before and after BC?’ ‘What changes did you notice in your sexual life and sexual health after BC?’ ‘What is your attitude about sexuality after BC?’ ‘What flaws do you experience about sex and sexual behaviors with your husband after BC?’ ‘Do you think that you have been able to meet your husband’s sexual expectations after BC?’.

The participants’ responses were considered for devising the next interview questions. Additional and probing questions were asked to improve the interviews’ depth as follows: ‘will you please explain it more?’ and ‘can you provide an example?’. The interviews lasted an average of 60 to 90 min. Seven participants were interviewed twice and for the rest was in one session. Therefore, 28 interviews were carried out with 21 participants. All interviews were audio-recorded applying a digital audio-recorder to ensure data accuracy.

A demographics questionnaire was also used to collect information about the women’s age, their husbands’ age, duration of marriage, education level, place of residence, number of children, employment status, economic status, time passed from BC diagnosis, and cancer-related treatments.

### Data analysis

The interviews were immediately transcribed *verbatim* by the first author (MM), and underwent data analysis by the research team (MM, AM, MG, MV) using the content analysis method concurrently with the data collection [22, 27]. Therefore, the transcripts were read line-by-line and several times to immerse in the data and to achieve a better understanding of the interviews’ content. Next, meaning units as parts of the interview sentences were identified, and was labeled during the open coding process. Similar emerging codes were grouped into the same category that were compared together and in relation to the entire data set in order to develop sub-themes and themes [28].

### Trustworthiness of data

Several strategies were used to ensure the trustworthiness of data [29]. Considering the inclusion criteria, 21 participants were recruited with a maximum variation in terms of age, occupation, gender, level of education, and family status. To ensure reflecting the participants' perspectives in the findings, the interviewer tried to bracket her own presumptions on the study phenomenon through writing reflexive notes. The research team worked together and had discussions to ensure the quality and validity of the data analysis process. A third person’s opinion was sought to remove ambiguities and resolve disagreements that emerged. As member checking, a brief report of the findings was given to 3 participants to ensure that our findings reflected their perspectives and experiences. Two qualitative researchers who were not the members of our research team confirmed our data analysis process as peer checking [29].

## Results

The mean (SD) age of the participants was 44.04 (SD = 4.29) years. All participants were married and their husband age was 49.85 (SD = 4.48) years indicating that the participants’ husbands were also at an age when sexual issues were important to them. The average duration of their marriage was 21.33 (SD = 6) years. Majority of the women had an under diploma education level (42.9%), were resident of the city (66.7%) and housekeeper (71.4%). Also, 66.8% of them had 1–2 children and 61.9% reported their economic status as relatively sufficient. BC in 42.9% of the participants were diagnosed in the last 1–3 years and in 57.1% of them were diagnosed more than 3 years ago. All participants had a history of chemotherapy, 61.9% had a history of radiotherapy, and 90.5% had undergone mastectomy (Table 1). Also, none of the participants underwent any breast reconstruction surgery.

**Table 1:** The participants’ demographic data

Variable	Mean (SD)
Age, range (y)	33–50
Women age (y)	44.04 (4.29)
Husbands age (y)	49.85 (4.48)
Duration of marriage	21.33 (6.00)
*Education level*	*N* (%)
Under diploma	9 (42.9)
Diploma	8 (38.1)
Academic	4 (19)
*Place of residence*	
City	14 (66.7)
Village	7 (33.3)
*Number of children*	
No children	2 (9.6)
One child or 2 children	14 (66.8)
Three or more children	5 (23.8)
*Occupation*	
Housekeeper	15 (71.4)
Employed	6 (28.6)
*Economic status (self-report)*	
Sufficient	3 (14.3)
Relatively sufficient	13 (61.9)
Not-sufficient	5 (23.8)
*Time passed from cancer diagnosis*	
1–3 years	9 (42.9)
More than 3 years	12 (57.1)
*Mastectomy*	
No	2 (9.5)
Partial	7 (33.3)
Total	12 (57.1)
Chemotherapy	21 (100)
*Radiotherapy*	
Yes	13 (61.9)
No	8 (38.1)

**Fig. 1:**
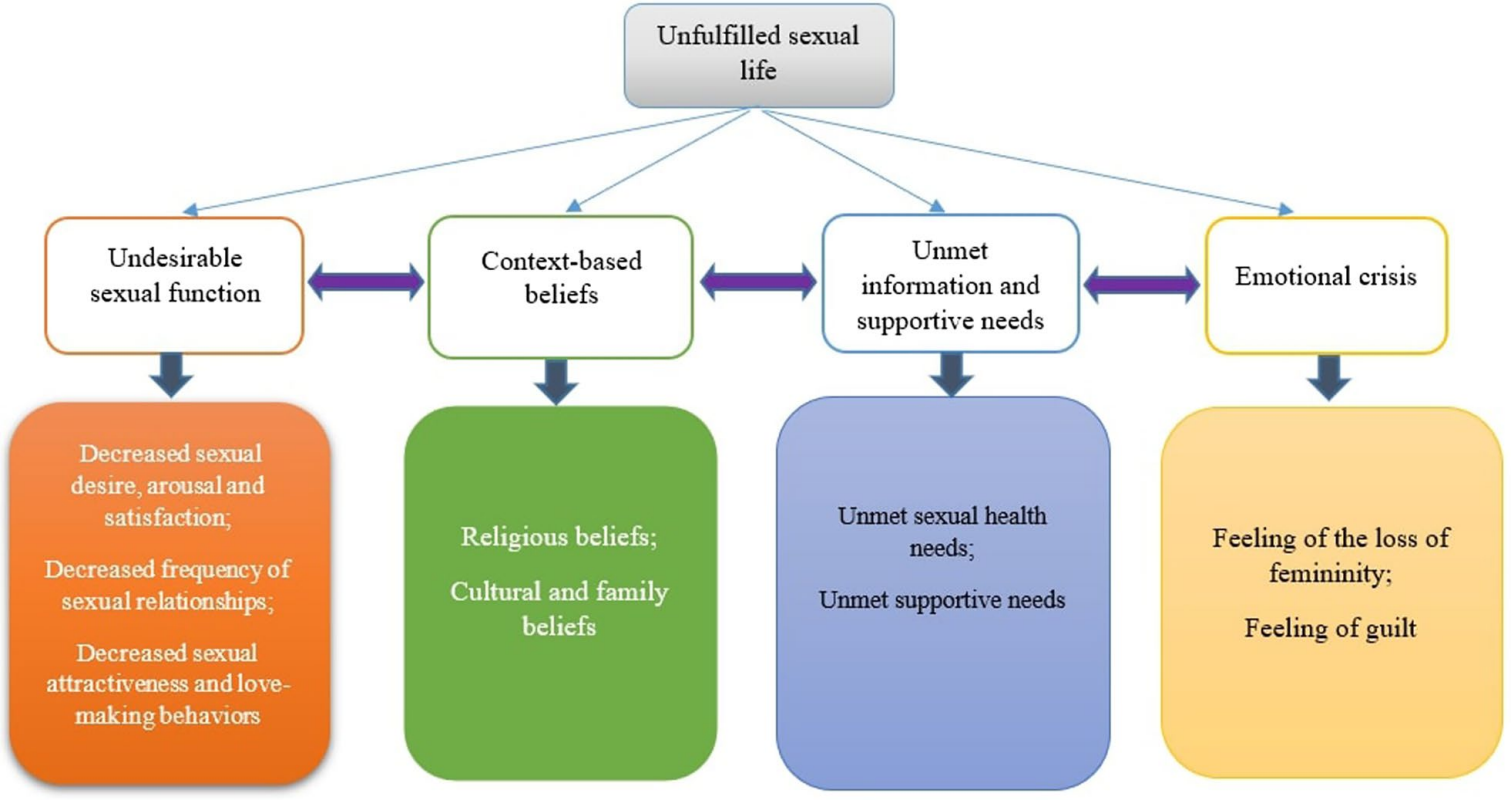
A summary of themes, subthemes and key codes developed in this study

The participants experienced changes in their sexual function due to BC that affected their sexual life. Their experiences were reflected in one main theme as follows: ‘unfulfilled sexual life’. Also, four subthemes were: ‘undesirable sexual function’, ‘context-based beliefs’, ‘unmet information and supportive needs’, and ‘emotional crisis’ (Fig. 1).

### Unfulfilled sexual life

Sexual life and sexual functioning were described as the main domains of health-related QoL among the BC survivors at reproductive age. However, due to physical and psychological changes raised by BC trajectory, unmet supportive needs by their spouses and healthcare systems, and the influence of beliefs originating from the social context, the BC survivors experienced unfulfilled sexual life. Factors influencing the feeling and experience of ‘unfilled sexual life’ by the participants were reflected in the subthemes of ‘undesirable sexual function’, ‘context-based beliefs’, ‘unmet information and supportive needs’, and ‘emotional crisis’. They were described with more details using direct quotations from the participants, as follows.

### Undesirable sexual function

Uncomfortable life conditions after BC treatments not only caused changes in the women’s sexual desire and arousal, but also had a negative impact on the sexual desire among their spouses. Furthermore, sexual intercourse did not necessarily lead to the optimal pleasure and the participants’ abilities to achieve optimal orgasm during sexual intercourse decreased. The reduction of sexual desire and being aroused, vaginal dryness, and dyspareunia were mentioned as influencing factors.

Some participants tried to abstain from sexual intercourse, but some were engaged in sexual activities just to please their husbands.

Although I did not have much sexual desire before BC, it reduced more after treatment, and I could say that my sexual desire really decreased very much. My husband's sexual desire was much higher than mine before BC, but his desire decreased also, because of interruptions in our sexual relationships for almost a year due to the BC treatment process. (Participant 2, 48 years old).

I feel like my body is a rock. Before BC, as soon as my husband had touched me, I was drowning in lust, but now it takes much time to find a desirable feeling to start sexual intercourse. (P 9, 37 years old).

Unpleasant! Before the disease, I used to enjoy sexual activity very much, and now have no much physical or psychological pleasure from it and even barely do it. This has affected my identity as a woman. (P 6, 47 years old).

Before my illness, I almost reached the orgasm during sexual intercourse, but after that, I never enjoyed it and felt bored. My vagina is dry, and I often want my husband to finish sexual intercourse quickly, because it is not pleasurable for me. (P 8, 42 years old).

Some participants thought that the loss of being aroused in breasts due to BC treatment especially mastectomy had a negative impact on the onset of as well as the continuity of sexual intercourse.

My breasts were very important to me during sexual intercourse so that to start having sex, my husband should touch and massage my breasts to get ready for it, but now that I do not have breasts, it is difficult to start sexual relationships. If my husband could suck my breasts during sexual intercourse, it is a great pleasure. (P21, 44 years old).

In contrast, two participants reported that having sexual relationships was a practical way of feeling alive after BC treatment and engaged them to have sexual relationship. Although it was the perspective of the minority, it meant that the adverse effects of BC on sexual relationship should not be imagined similarly in all participants.

After treatments, I feel that I am more interested in sexual relationship than before. I wanted to have sexual relationship with my spouse and show him that nothing has been changed compared to the past. (P 19, 40 years old).

Most participants experienced a decrease in the frequency of sexual relationships and sexual love-making behaviors in married life due to unhappiness from physical and psychological changes raised from BC and its treatments including scars on the breast, hair loss, and weight gain. A reduction in sexual love-making behaviors between spouses led to dysfunction in sexual arousal, sexual desire, and eventually the decrease of sexual function. Some participants expressed that they only engaged in sexual intercourse two or three times per year.

The appearance of my breasts has been completely ruined, because of surgeries and radiotherapy around my breasts. It is still different from other parts of my body, which has affected my self-confidence and I think I do not have the same charm, freshness, and vitality for my husband as before. So that his requests for sexual relationships have decreased. Women enjoy their appearance and try to show it to their spouses. (P 6, 47 years old).

I hate to look at myself in the mirror and I can't even touch my breast or let anyone look at it or touch it. (P 12, 44 years old)

The clothes I wear to go out for shopping or at work, should be loose and not to show that I have no breasts, but during sexual relationships when I am naked in front of my husband, I am embarrassed and feel bad … therefore, I often do not like to have sexual activities. (P11, 33 years old).

During the treatments, I had no sexual relationships for 9 months and my vagina became dry and I was taking vitamin E. Previously, I had sexual relationships two or three times a week, but now it is maybe one time per month. (P 2, 48 years old).

Before getting sick, I loved to be hugged, kissed, and cuddled. After my illness, all of these subsided. Neither my husband nor I have enough patience to do these anymore. (P 4, 44 years old).

…after BC, even I hated to be hugged anymore. I do not want to do these without having hair and breasts. Maybe one of the reasons is that my husband is no longer willing to do such sexual behaviors. (P 20, 38 years old).

### Context-based beliefs

Some participants expressed that religious beliefs encouraged them to obey their husbands and enter a sexual relationship with them, even if they felt unwell, because of the disease.

I care a lot about religion. God says that if my husband sleeps unhappy at night, the angels will curse me. I say to God, ‘I do not want such a thing to happen’…God's pleasure is so important to me; therefore, I am ready to do anything and that's why I do not neglect having sexual relationships with my husband. (P 17, 49 years old).

Because God commands that a woman should satisfy her husband, I allow him to have sexual intercourse with me whenever he asks for it. (P 20, 38 years old)

Moreover, the participants stated that cultural beliefs in terms of the presentation of requests for having sexual relationships by the husband and shame and modesty of women to start sexual relationships had negative impacts on their sexual function and adversely made them not to express their willingness to have sexual intercourse. So, these underlying beliefs prevented them to start a sexual relationship.

Before my disease, I was not asking for sexual relationships. I was feeling that the request should be one-sided and would be presented by my spouse. After BC, although I tried to have a better relationship by talking and love making, my shame and modesty made me not talk about my sexual needs with my spouse. (P 3, 46 years old).

I can never tell my husband to have sexual relationships with me … not at all … I will not do this even if I die. If I do this, he will think to himself that I am shameless … our marriage life will be over…. We have three children who are in the marriage time, and we avoid having sexual-related behaviors in front of them. (P 4, 44 years old).

Furthermore, the participants highlighted that family beliefs such as their own or their husbands’ parents’ cool romantic behaviors affected their marriage and sexual life even after completing BC treatments.

My parents were very warm and had a romantic relationship with each other and I always wanted to be like them. But my husband's parents were cold and fighting each other sometimes. In their family, it was a custom that when children get a little older, parents slept separately. My husband and I follow this tradition after my disease. It was mostly, because of my illness … but the behavior of my husband's parents affected it. (P 6, 47 years old).

I do not tell my husband now that I love kissing and hugging, because of my family. For example, I never saw my parents kiss each other … I feel that the cool romantic behavior of my parents has affected me … after my disease, we do not have romantic behaviors at all. (P 2, 48 years old).

### Unmet information and supportive needs

The BC survivors did not receive any information from healthcare providers regarding sexual issues and sexual complications caused by induced menopause, vaginal dryness, sexual frigidity, and how to deal with them.

When my first chemotherapy session was over, my menstruations stopped, but I did not receive any information that chemotherapy could cause the cessation of menstruation. I was stressed out. (P 20, 38 years old).

I was so upset, because I did not know what would happen in the future. After the chemotherapy, I had sexual relationships time to time, my vagina was very dry, and no one told me that I would experience this problem and what I should do to deal with it. (P 11, 33 years old).

In my culture, sexual issues are ignored, and people cannot easily raise their sexual problems. Even in hospitals, education need about sexual issues is not addressed. (P19, 40 years old).

This is ruinous and a complete shock; no one says that cancer treatments may ruin the sexual life. (P18, 50 years old)

In addition, some participants reported that they had no information about the complications of BC treatment, recurrence and exacerbation of BC symptoms. This lack of information made them to experience a fear and eventually led to the coldness of sexual relationships.

I was scared during the sexual intercourse that if I got pregnant, my child would be disabled due to medications’ side effects. Due to such a fear, I was getting cold in my sexual relationships. (P 11, 33 years old).

It is painful to think that I undertake surgery in my breasts. When I feel pressure in my breasts, even if they are not painful, I am scared that my condition may get worse. I felt more comfortable before the disease. (P 2, 48 years old).

Furthermore*,* the supportive needs of most BC survivors were not met by their spouses after the diagnosis and completion of BC treatment, which led to emotional separation and damaged their romantic and sexual behaviors.

I went to another city for radiotherapy where my brother's house was there. My husband sent me there with my son. I was there for a month, the distance between that city and our city was about 250 km, and my husband did not even come to see me once. I felt that I was an extra load and it made me move away little by little. This feeling influenced our marital relationships. (P 11, 33 years old).

My husband did not leave me alone after my illness, but the change in his behaviors was noticeable. He was no longer the person I knew in the past, and was not taking care of me as before. I felt lonely. (P 12, 44 years old).

In contrast, three participants stated that they received more support by their husbands that led to greater intimacy with them after the diagnosis and treatment of BC.

My husband frequently tells me that ‘you are important to me and it does not matter at all that you do not have breasts, and in any case, I love you’. I actually feel this disease has made me appreciate my husband's feelings and have more intimacy toward each other. (P 3, 46 years old).

### Emotional crisis

The participants reported the presence of emotional crisis affecting their marital life and sexual behaviors. Physical changes such as mastectomy and the cessation of menstruation caused the feeling of loss of femininity among the participants, which impaired their sexual desire and function.

I have a son, but I would like to have another child. My husband is young, and he may want another child, and I may not be able to have a baby for several years. I feel empty, because I can't get pregnant like a healthy woman. However, the doctor said that I could have a baby after five years. One of the reasons why my sexual desire has decreased is that I don't have the fertility power anymore. (P11, 33 years old).

Although I had no menstruation for about a year before chemotherapy and didn't even remove my breast completely, I feel that I am an imperfect woman, because my breast has become smaller and I feel an imperfect woman. (P18, 50 years old).

Moreover, five participants believed that their husbands were oppressed, because they suppressed their sexual needs due to BC. So, they blamed themselves and felt guilty and entered sexual relationships with their husbands to alleviate their feeling of guilt.

Sometimes I tell him that I'm bored right now, and we cannot have sexual relationships, but because I feel that he is suppressing his own sexual needs because of me, I convince myself to have sexual relationships. (P 9, 37 years old).

I'm worried about whether my husband's sexual needs are met. Sometimes I have sex with him only because of his sexual needs, and it is against my will. (P10, 44 years old)

## Discussion

Sexuality and sexual well-being among BC survivors at reproductive age have been studied by a few qualitative studies. Therefore, this qualitative descriptive research aimed to explore the experiences of BC survivors at reproductive age regarding their sexual life and related issues in an urban area of Iran. Our findings highlighted the significance of the sexual and marital life among the BC survivors and indicated what changes happened to their sexual health following BC treatments. The developed theme and subthemes have been discussed through the comparison of our findings with those of similar studies in other contexts, as follows.

The participants experienced undesirable sexual function after BC consisting of changes in sexual desire, decreased sexual satisfaction, and decreased sexual arousal. Similarly, a qualitative study by Wang et al., on twenty Chinese women suffering from BC showed that the majority of women with BC suffered from a lack of sexual desire [21]. In addition, association between the loss of sexual desire and satisfaction with mastectomy due to BC was depicted by Turkish women with BC through a cross-sectional study by Özturk and Akyolcu [30]. Cairo Notari et al., in a qualitative study of the French women's experiences of sexual functioning in the early weeks of BC treatments showed that most women experienced a lack of sexual desire [31]. Moreover, the results of a systematic review demonstrated that women with BC experienced some degrees of sexual issues including decreased sexual arousal, orgasm, libido, and sexual pleasure [32].

The decreased frequency of sexual relationships, decreased sexual love-making behaviors, and sexual unattractiveness were stated as other aspects of undesirable sexual function by the BC survivors. Consistent with our findings, the decreased frequency of sexual relationships in patients with BC has been reported in different contexts. For instance, Wang et al., showed that almost all patients reported a significant reduction in the frequency of sexual activities [21]. Moreover, Leila et al.’s study on fifty Tunisian women with BC reported that more than half of them after cancer experienced a reduction in the frequency of sexual intercourses and reported sexual satisfaction [33]. A systematic review by Chang et al., demonstrated a reduced frequency of sexual relationships in women with BC after treatment [15].

Furthermore, difficulties in sexual life consisting of posttreatment disturbances in body image and feelings of unattractiveness seem to be highly prevalent among BC survivors [34]. Consistent with our results, a qualitative study on Nigerian women after mastectomy demonstrated that mastectomy had a remarkable influence on the women’s perceptions of femininity and reduced their sexual function [35]. A phenomenological study in Jordan showed that many women with BC were dissatisfied with their appearance and particularly were sensitive regarding their sexual unattractiveness due to cancer treatments. They feared that their appearance might damage their relationships with their husbands who no longer found them attractive [36]. Rezaei et al., in a comprehensive literature review documented that disturbances in physical appearance arising due to cancer treatments among BC survivors had destructive effects on couples’ marital relationships [37].

It was found that context-based factors including religious, cultural, and family beliefs played important roles in the women’s sexual relationships with their spouses, so as it encouraged the participants or hindered them to have sexual intercourse. Similarly, a qualitative study on eighteen women with BC in Saudi Arabia showed that belief in God and following God's commands were reflected in the feeling of a duty to please their husbands. Therefore, they decided to continue their marital relationships and lifestyle based on previous life routines [38]. A systematic review by Chang et al., concluded that religious beliefs were inseparable from sexuality and affected the reactions of patients with BC to sexual issues [15]. A qualitative study on African American women with BC in the rural regions of Eastern North Carolina reported that religious beliefs and faith in God were the most important defense mechanisms influencing participants’ relationships in BC trajectory [39]. In another study on 35 Palestinian women with BC it was found that religion and faith were key elements to accept BC as a destiny from the point of Muslim and Christian participants [40]. Given differences in the cultural and religious context in different countries [41], healthcare providers need to consider cultural and religious differences during the provision of care to these patients [38].

The study findings revealed that the sexual health information and supportive needs of the BC survivors had not been adequately met, which impacted their sexual relationships. Similar to our findings, the Klungrit et al.’s study on Thai women with BC showed the presence of a need for family support and education by nurses and physicians about the effects of BC and the side effects of chemotherapy and radiotherapy [42]. Also, in the study of Den Ouden et al., the majority of BC patients in the Netherlands expressed the need to receive information about the effect of BC on sexuality and intimacy from nurses or physicians [43]. In addition, two review studies by Hill et al., and Bartula and Sherman, indicated the need to pay more attention to sexual concerns in patients with BC as an unmet need [44, 45]. In contrast, the Hammoudeh et al.’s study showed that the husbands of patients with BC accepted physical changes associated with the disease in their wives including having no breasts and therefore, provided them with emotional support [40]. Talking about sexual issues is taboo and women often refuse to talk about their sexual problems that leads to not meeting their information needs. On the other hand, healthcare professionals may ignore patients’ need for education about sexual issues for a number of reasons including lack of awareness, uncomfortable feelings, lack of education, privacy of sexual matters, lack of a private environment, lack of an appropriate referral system, and heavy workloads leading to the lack of time to provide information [43].

The participants experienced emotional crisis indicating the feeling of loss of femininity and feeling of guilt, which influenced their sexual behaviors. Also, the feeling of guilt in the participants raised from their concerns about the sexual unmet needs of their husbands encouraged them to continue sexual relationships with their husbands. The women’s perception of attractiveness and femininity greatly depends on the their physical features such as body figure, facial appearance, hair color and length, and breast size and form, which are negatively affected by BC trajectory [46, 47]. A qualitative study in Turkey by Koçan and Gürsoy on women with BC who were undergone mastectomy reported that mastectomy adversely affected the feelings of femininity [48]. This suggests that healthcare providers should be especially sensitive to the consequences of BC on the women’s feeling of femininity. In addition, another study in the United States by McClelland showed that women with BC experienced a feeling of guilt, because they did not respond appropriately to their husbands’ sexual intimacy [49].

## Limitations

The current study is one of the few qualitative studies, if not the first one, on the sexual life of Iranian BC survivors. Applying different trustworthiness approaches in data collection and analysis are the strengths of this qualitative study, which have increased our trust in the quality of our findings. Therefore, our findings can inform healthcare providers about sexual concerns among BC survivors. This study was conducted only in a hospital in an urban area of Iran that might impact on the transferability of our findings to other contexts. However, it contains practical implications for patient care in other Muslim countries. It was also difficult to reach a deep understanding of the participant’ sexuality life due to taboo attached to it in the Iranian context and the fear of rupture of the alliance between the participants and the researcher.

## Conclusion

Our study highlighted that Iranian BC survivors at reproductive age following BC treatments experienced ‘unfulfilled sexual life’. This research presents concerns and concepts that can be used for designing future qualitative and quantitative studies aiming at mitigating sexuality and marriage issues among BC survivors. The contexed-based identity of our findings affecting sexual life among BC survivors should be considered during their interpretation. Nurses and other healthcare providers should be aware of changes in the sexual life of BC survivors. They should cooperate in multidisciplinary teams to evaluate sexual life disturbances and initiate timely interventions and support. It is suggested to establish sexual health counseling units for BC patients and survivors in healthcare systems to aid this vulnerable group of women and improve their long-term sexual satisfaction. Also, future research should consider the development of useful practical instruments for the assessment of sexual life among BC survivors.

## Supplementary Information

**Additional file 1.** The interview guide used for the data collection in this study.

## Data Availability

Data is available from the corresponding author on reasonable request.

## Abbreviations

BC: breast cancer
QoL: quality of life
SRQR: standards for reporting qualitative research
WHO: world health organization
