# Supplementary material for: Qualitative exploration of sexual life among breast cancer survivors at reproductive age
Source: BMC Womens Health. 2021 Feb 9;21:56. doi: 10.1186/s12905-021-01212-9 (PMC7871536; doi:10.1186/s12905-021-01212-9)
Supplement: Supplementary file 1 — Additional file 1. The interview guide used for the data collection in this study. [file 12905_2021_1212_MOESM1_ESM.docx]

**Interview guide**

- How have your sexual experiences changed before and after BC?
- What changes did you notice in your sexual life and sexual health after BC?
- What is your attitude about sexuality after BC?
- What flaws do you experience about sex and sexual behaviors with your husband after BC?
- Do you think that you have been able to meet your husband's sexual expectations after BC?

Probing questions

- Will you please explain it more?
- Can you provide an example?
